# Factor Structure and Measurement Invariance Across Gender of the Beck Depression Inventory-II in Adolescent Psychiatric Patients

**DOI:** 10.3389/fpsyt.2020.527559

**Published:** 2020-12-23

**Authors:** Ferdinand Keller, Inken Kirschbaum-Lesch, Joana Straub

**Affiliations:** ^1^Department of Child and Adolescent Psychiatry and Psychotherapy, University of Ulm, Ulm, Germany; ^2^LWL-University Hospital for Child and Adolescent Psychiatry and Psychotherapy, Ruhr-University Bochum, Hamm, Germany

**Keywords:** beck depression inventory, factor structure, clinical sample, adolescents, gender, bifactor models, unidimensionality

## Abstract

The revised version of the Beck Depression Inventory (BDI-II) is one of the most frequently applied questionnaires not only in adults, but also in adolescents. To date, attempts to identify a replicable factor structure of the BDI-II have mainly been undertaken in adult populations. Moreover, most of the studies which included minors and were split by gender lacked confirmatory factor analyses and were generally conducted in healthy adolescents. The present study therefore aimed to determine the goodness of fit of various factor models proposed in the literature in an adolescent clinical sample, to evaluate alternative solutions for the factor structure and to explore potential gender differences in factor loadings. The focus was on testing bifactor models and subsequently on calculating bifactor statistical indices to help clarify whether a uni- or a multidimensional construct is more appropriate, and on testing the best-fitting factor model for measurement invariance according to gender. The sample comprised 835 adolescent girls and boys aged 13–18 years in out- and inpatient setting. Several factor models proposed in the literature provided a good fit when applied to the adolescent clinical sample, and differences in goodness of fit were small. Exploratory factor analyses were used to develop and test a bifactor model that consisted of a general factor and two specific factors, termed cognitive and somatic. The bifactor model confirmed the existence of a strong general factor on which all items load, and the bifactor statistical indices suggest that the BDI-II should be seen as a unidimensional scale. Concerning measurement invariance across gender, there were differences in loadings on item 21 (Loss of interest in sex) on the general factor and on items 1 (Sadness), 4 (Loss of pleasure), and 9 (Suicidal Thoughts) on the specific factors. Thus, partial measurement invariance can be assumed and differences are negligible. It can be concluded that the total score of the BDI-II can be used to measure depression severity in adolescent clinical samples.

## Introduction

The Beck Depression Inventory (BDI) was developed in 1961 for the assessment of depressive symptoms and was subsequently revised in 1996, leading to the revised version of the Beck Depression Inventory (BDI-II) (1). It is one of the most frequently applied questionnaires not only in adults, but also in adolescents between the ages of 13 and 18 years (2). The BDI-II measures symptoms of depression severity on the behavioral, emotional, cognitive and somatic level by summing ratings of all 21 items on a 4-point rating scale (0–3). In line with the criteria for depressive disorders in the International Classification of Diseases (ICD-10) and the Diagnostic and Statistical Manual of Mental Disorders (DSM-IV and also in the DSM-V), symptoms are assessed over the past 2 weeks.

### Exploratory Factor Analyses in Adult Samples

To date, attempts to identify a replicable factor structure of the BDI-II have mainly been undertaken in adult populations. In general, the psychometric properties of the BDI-II have been shown to be very good. Results regarding factorial validity were presented for the first time in the original manual (1) and were based on data from 500 adult psychiatric outpatients. These results suggested the existence of two factors, “somatic-affective” and “cognitive.” Although Steer et al. (3) were able to replicate this distinction, other studies were not. A recent meta-analysis (4) evaluated all studies published in the English language. Concerning the full sample, exploratory factor analyses found the two-factor solution (37 out of 56 studies), with one “cognitive factor” and one “somatic-affective factor,” to be the most acceptable one. The two-factor structure was also supported for subgroups of studies: the factors “negative attitudes” and “somatic-affective” were the best-fitting with respect to [1] the English-language version of the BDI-II, [2] clinical populations and [3] adult populations. Concerning [1] non-English versions of the BDI-II, [2] non-clinical samples, and [3] youth/college students, the “cognitive” and “somatic-affective” factors seemed to be the most representative. In their second meta-analysis, Huang and Chen (4) aggregated 16 independent samples, providing the intercorrelation matrix among the BDI-II items. The authors found that besides the two-factor solution, the existence of one general depression factor was also supported by the good fit of the one-factor model.

### Confirmatory Factor Analyses in Adult Samples

In contrast to the *simple structure* models revealing the above-mentioned factor solutions, some authors have put forward a bifactor model with respect to the BDI-II, which constitutes a special case of a *complex structure* model. The difference between simple models and bifactor models lies in the assumption of a general factor on which all items load, and two or more group factors on which only some items load (5, 6).

Ward (7) conducted confirmatory factor analyses with six data sets from five previously published studies (three clinical and three college samples) in order to compare the frequently published two-factor structure, with a bi-factor model consisting of a general factor and two orthogonal specific factors, and concluded that the bifactor model fits as well or better than the two-factor models. This bifactor model also was found among the best fitting models in a Dutch sample of outpatients with mixed psychiatric diagnoses (8). Another study (9) analyzed the factor structure of the BDI-II in adult inpatients diagnosed with a primary affective disorder, and concluded that the BDI-II is best represented by four factors: a general factor, a cognitive factor, a somatic factor and an activation factor. This model, derived from non-metric multidimensional scaling (10), even exceeded the fit of the bifactor model of Ward (7), and the improvement in goodness of fit was replicated in a second sample of inpatients with depression (9). A bifactor model with a general depression factor and three specific factors (cognitive, affective and somatic) was found in a large mixed (clinical and non-clinical) sample from the Dominican Republic (11).

This bifactor structure was also found to have the best fit out of 15 competing factor models in a sample of outpatients with depressive disorder and adjustment disorder (12). However, high factor loadings on the general factor and inspection of indices for explained variance by the factors led the authors to conclude that the BDI-II measures a single latent construct, but that it may be useful to use the subscale scores in combination with the total score for treatment decisions. Similarly, Lim et al. (13) argued for a mainly unidimensional depression factor based on their results in a sample of Korean adults, in which they found a strong general factor and two specific factors, termed somatic and cognitive.

### Exploratory Factor Analyses in Adolescent and Young Adult Samples

However, there are fewer studies with respect to the factor structure of the BDI-II in adolescent/young adult samples (age between 12 and 20 years). Most of the existing studies were conducted in healthy students (14–22), some examined outpatients (21, 23–25) and one study examined an inpatient sample (26). In the examined clinical samples, most participants fulfilled criteria for various psychiatric disorders. Most of the studies that ran an exploratory factor analysis (EFA) or principal component analysis (PCA) revealed a two-factor solution (15, 17–21, 25, 26). In most of the two-factor studies, the first factor was called either “cognitive,” “cognitive-affective,” or “cognitive-somatic,” and the second factor either “somatic-non-specific,” “somatic,” or “somatic-affective.” The three-factor solution found by Steer et al. (23) was ultimately considered as a two-factor solution (for details, see below in Method section). One study did not support any clear factor structure (24).

A model with three factors was developed by Byrne and colleagues in a series of studies, mainly in the context of non-clinical adolescent samples and subsequently tested in several countries (27), e.g., for the Chinese version of the BDI-II (14). The three factors were “negative attitude,” “performance difficulties,” and “somatic elements.” Due to the substantial correlation between the three factors and the theoretical justification of a general depression construct, Byrne et al. (14) see also (27) suggested a second-order factor structure. Wu and Huang (28) suggested the same three factors, but without the second-order factor structure (see Method section for detailed information), and this three-factor structure was replicated in a non-clinical adolescent sample (22).

### Confirmatory Factor Analyses in Adolescent Samples

To date, only a small number of studies have conducted confirmatory factor analysis in minors, especially in clinical samples. Only one study (26) conducted a CFA with respect to data from 408 adolescent psychiatric inpatients in order to examine the adequacy of fit of previously defined first-order factor solutions: (a) the two-factor solution reported in the BDI-II manual (1), (b) the two-factor solution reported by Dozois et al. (29) for college undergraduates, (c) the three-factor solution reported by Steer et al. (23) for adolescent psychiatric outpatients, and (d) a one-factor solution. The authors concluded that none of the models met all of the pre-established initial and final adequacy-of-fit criteria. Subsequent EFAs were conducted to explore alternative solutions of the BDI-II items, and the authors identified two factors: The first factor contained all nine items of the original cognitive factor reported in the BDI-II manual (1) and the second factor contained eight items that were similar to the original 12 somatic-affective factor items. Only one item (10, “crying”) failed to load on either of these factors.

In a further study the fit estimates of the factor solutions stated above were tested in a non-clinical adolescent sample (16), finally retrieving the two-factor model that was reported in their previous study (26). Furthermore, the authors applied a bifactor model to the best-fitting model for their study sample data. The results revealed stronger support for the bifactor model than for the two-factor model, revealing one general factor and two specific factors, “somatic” and cognitive-affective.” Lee et al. (22) also tested several existing factor models and found the three-factor model of Wu and Huang (28) to be the best-fitting model.

### Differences Between Boys and Girls Regarding the Factor Structure

Given the differences in the manifestation of depressive symptoms in girls and boys, e.g., girls show higher prevalence rates and more internalizing behavior than do boys [e.g., (30, 31)], it appears to be fruitful to compare the factor structure of the BDI-II between genders. To date, only four studies have investigated the factor structure of the BDI-II in adolescents split by gender. Three of these studies included healthy adolescents (15, 18, 19) and one included psychiatric inpatients (26). Two studies applied PCA (15, 18) and two applied EFA (19, 26). All of the studies supported the two-factor model, with the first factor being called “cognitive-affective” in all four studies and the second factor “somatic-non-specific” (15, 18, 19) and “somatic-affective” (26). Split by gender, certain items that loaded on factor one in girls did not load on the same factor in boys. This was also the case for the second factor, e.g., “loss of interest” and “loss of energy” loaded on the factor “cognitive-affective” in girls and on the factor “somatic-affective” in boys (26).

While these exploratory studies provided some insight into potential differences in factor structure between boys and girls, they did not rigorously test the comparability of the factor solutions, i.e., whether BDI-II scores measure the same constructs in the same manner across boys and girls. Wu and Huang (28) tested the gender-related measurement invariance of the BDI-II in a sample of Taiwanese adolescents. The authors found that measurement invariance was established at the level of configural, metric, and partial scalar invariance (seven non-invariant intercepts for the items 2, 3, 7, 9, 10, 12, and 19 were identified). Thus, factor loadings can be considered as equal across gender groups, but latent means may be affected by the non-invariant intercepts (28).

### Aims of the Present Study

Taken together, studies that aimed to investigate the factor structure of the BDI-II by means of simple and complex factor models revealed inconsistent results in terms of the number of factors and their nomenclature, respectively. Most of the studies which were conducted with minors and were split by gender lacked confirmatory factor analyses and usually included healthy adolescents. Therefore, an examination of the BDI-II factor structure in adolescent clinical samples is warranted. The bifactor approach was preferred, since all more recent factor analyses revealed that a bifactor model had a better fit compared to first-order factor models (8, 9, 12, 16). The aims of the following study were [1] to determine the goodness of fit of various factor models proposed in the literature in a clinical sample of adolescents, [2] to evaluate alternative solutions for the factor structure for the whole sample and to explore potential gender differences, with a focus on testing bifactor models and subsequently on calculating the ratios of variance explained by the general and the specific factors to help clarify whether a uni- or a multidimensional construct is more appropriate, and [3] to test the best-fitting factor model for measurement invariance according to gender.

## Materials and Methods

### Sample

The sample consisted of three subsamples recruited from the Department of Child and Adolescent Psychiatry and Psychotherapy, University of Ulm (*N* = 548 outpatients and *N* = 112 outpatients and inpatients) and from the LWL-University Hospital of Child and Adolescent Psychiatry and Psychotherapy Hamm, Ruhr-University Bochum (*N* = 175 inpatients). The psychometric properties of the BDI-II in the mixed sample from Ulm (*N* = 112) have previously been published elsewhere (24). The BDI-II was completed in the course of the routine diagnostic assessment at the patients' first visit to the respective clinic. Inclusion criteria were an IQ ≥ 80 and age between 13 and 18 years. Of the total sample (*N* = 835), 490 adolescents (58.7%) were females and the mean age was 15.79 (SD = 1.38). There were no significant age differences between boys (*M* = 15.74 years, *SD* = 1.46) and girls (*M* = 15.81 years, *SD* = 1.33), *t*(833) = −0.73, *p* = 0.464. The mean IQ was 100.92 (SD = 12.68). Concerning ICD-10 diagnosis, 471 (56.4%) fulfilled criteria for depressive disorder (F32 depressive disorder; F33 recurrent depressive disorder; F41.2 mixed anxiety and depressive disorder; F92.0 depressive conduct disorder). Following the first visit, 65.6% were treated in the outpatient setting and 34.4% in the inpatient setting.

### Measure

The BDI-II consists of 21 items that are answered on a scale ranging from 0 to 3. Each category has an item-specific text. The scale yields a total severity score ranging from 0 to 63. The revised manual suggests that depression scores should be categorized as minimally (0 to 13), mildly (14 to 19), moderately (20 to 28) and severely (29 to 63) depressed. Internal consistency ranged between 0.89 ≤ α ≤ 0.94 in psychiatric samples and between 0.84 ≤ α ≤ 0.91 in non-psychiatric samples. Test-retest reliability and validity were also reported as high (1, 32).

### Statistical Approach and Model Estimation Procedures

Since there are several factor models reported for the BDI-II in adolescents (see overview for the models tested with CFA below), in a first step, we tested the goodness of fit of these models when applied to our data. In addition, we included some models from research in adult samples, in particular the model of Ward (7), which can be considered as one of the best-fitting models across multiple studies, and a model proposed by Bühler et al. (9), which includes an additional third factor, termed activation factor. For the second aim, i.e., exploring alternative solutions, we began with an EFA for the whole sample and for boys and girls separately. Solutions from one to four factors were compared in order to find the best-fitting configuration for the whole sample and within each gender group, and to evaluate whether the number of factors and the patterns of indicator loadings on the factors remain the same. The best-fitting model (in terms of interpretability and goodness of fit) was then selected and further analyzed as a bifactor model, i.e., all items load on the general factor and, in addition, each item loads on the specific factor to which it most belongs.

For the third aim, i.e., to identify potential differences in factor structure between boys and girls and to determine measurement invariance, the candidate model from the second aim was used as the baseline model and a multi-group CFA was applied to determine the goodness of fit and the equivalence in the structure of factor loadings between gender groups (configural invariance). If configural invariance holds, the resulting factor solution is evaluated further for metric invariance, i.e., factor loadings are fixed to be equal in both groups. For ordered-categorical variables, these equality constraints imply that factor loadings and thresholds for a variable should be constrained in tandem (33), since they are dependent [note, however, that other authors, e.g., (34), or in the case of testing longitudinal invariance Liu et al. (35), split it into two steps]. Metric invariance was tested by fitting a multi-group CFA in which factor loadings and thresholds were constrained to be equal across groups; residual variances were fixed at one in the first group and freely estimated in the second group. The fit of this model was compared to the fit of the multi-group CFA of configural invariance using the appropriate Chi-square difference test (DIFFTEST) for nested ordered-categorical CFA models in the case of WLSMV estimation provided by M*plus* (33). If the difference was significant, i.e., the assumption of equal factor loadings and equal thresholds did not hold, partial metric invariance was examined by inspecting the items with substantial between-group differences in factor loadings. These items were set free across groups in factor loadings and in thresholds [but the residual variance was fixed at one for identification purposes, c.f. (33)] and the model fit was compared to the model with configural invariance. Throughout these analyses, local fit indices (residuals, modification indices) were also examined. Furthermore, the magnitude of the Chi^2^ value with the expected value of the sample distribution, i.e., the number of degrees of freedom (*df*), was used. For a good model fit, the ratio Chi^2^/*df* should be small. As no absolute standards have been defined, a ratio between 2 and 3 is indicative of a “good” or “acceptable” data model fit, respectively (36).

The fit of the models was evaluated using the comparative fit index (CFI), the Tucker–Lewis index (TLI), and the root mean square error of approximation (RMSEA). A CFI ≥ 0.95, an RMSEA value ≤ 0.06, and a TLI ≥ 0.95 are considered as indicating a good fit, according to the guidelines of Hu and Bentler (37). A reasonable fit is indicated for values of CFI ≥ 0.90, and RMSEA ≤ 0.08 (38, 39).

All factor analytic models were estimated using M*plus* version 7.4 (33). Items were treated as ordered-categorical and the mean and variance-adjusted weighted least squares (WLSMV) estimator was used. In all simple structure factor models, factors were allowed to correlate, whereas items were not unless otherwise indicated. For bifactor analyses, the intercorrelations between the general factor and the specific factors were all fixed to zero. Statistical indices to evaluate bifactor models (6), i.e., to separate and compare several sources of variance due to the general factor and to the specific factors alone, were coefficient omega and omega hierarchical, the concept of explained common variance (ECV), and the percent of uncontaminated correlations (PUC); all coefficients were calculated according to the formulas given in Rodriguez et al. (40).

### Overview of Factor Models Tested With CFA

Eight factor models were selected for testing the goodness of fit when applied to our data. Information about the original sample and the sample size is provided in **Table 2**.

The factor model of Steer et al. (3) consists of two factors; the cognitive factor includes items 2, 3, 5–9, and 14 (eight items), and the somatic-affective factor items 1, 4, 10–13, 15–21 (13 items).

Steer et al. (23) extracted a solution with three factors using a sample of 210 adolescent outpatients (50% female). The cognitive factor was defined by items 2, 3, 7–9, 13, 14, and 19 (eight items), and the somatic-affective factor by items 1, 4, 12, 15–18, and 20 (eight items). The third factor (guilt-punishment) consists of items 5, 6, and 10 (three items). As item 10 (crying) also loaded on the somatic-affective factor, and the guilt-punishment factor was composed of only three items, Steer et al. (23) did not consider this factor to be generalizable; furthermore, items 21 (loss of interest in sex) and 11 (agitation) did not load saliently on any of the three factors. However, following Osman et al. (16), the factor was included in the tested three-factor model of Steer et al. (23), and item 21 was added to the somatic-affective factor while item 11 was omitted, as performed by Osman et al. (16, p. 90).

In the model of Osman et al. (26), the cognitive-affective factor was composed of items 1–10 and 12–14, while items 11 and 15–21 defined the somatic factor (c.f. 16, p. 90).

The model developed by Byrne and colleagues in a Hong Kong sample (14, 27) consists of three factors: negative attitude (items 1–3, 5–10, 14), performance difficulty (items 4, 11–13, 17, 19) and somatic elements (items 15, 16, 18, 20). Note that item 21 (loss of interest in sex) is not included since the schools in Hong Kong objected to this item (for the current analysis, however, item 21 was added to factor somatic). Due to substantial correlations between the three factors and a theoretical justification of a General Depression construct, Byrne et al. [(14); see also (27)] suggested a second-order factor structure.

The factor model of Wu and Huang (28) relies on the same three factors as in Byrne's model, but without the second-order factor structure, and item 21 is included within somatic elements. Furthermore, their model includes three correlated item pairs (2 and 3; 4 and 12; 16 and 18) that were not modeled here.

Ward's (7) bifactor model includes a general factor and two orthogonal group factors. All 21 items load on the general factor, items 2, 3, 5–9, and 14 load on the cognitive group factor, and items 15, 16, 18–20 load on the somatic group factor. Two item pairs (7 and 8; 4 and 12) have correlated error terms.

The bifactor model of Osman et al. (16) also includes a general factor on which all items load, and two group factors (somatic with items 15, 16, 18–20, and the remaining 16 items on the cognitive-affective factor).

Bühler et al. (9) proposed a more general complex factor model with a general factor and three orthogonal group factors, where items are allowed to load on more than one group factor. All 21 items load on the general factor, items 2, 3, 5–9, and 14 load on the cognitive group factor, items 10, 11, 16–21 on the somatic group factor, and items 6, 9–11, 15, 17, 19, and 20 on the activation group factor.

## Results

### Descriptive Statistics and Gender Differences

The item-specific means, standard deviations and the item-total correlations are shown in Table 1 for the total sample as well as for boys and girls separately. Concerning the total sample, all item-total correlations were sufficient to good, with the exception of item 21 (loss of interest in sex), which also had the lowest mean value. The internal consistency estimate was high (Cronbach alpha = 0.94). The girls had consistently higher mean values than did the boys, and this difference was significant with at least *p* < 0.005 for every item. The difference in the total score was 10.1 and was significant [*t*(821.8) = 11.86, *p* < 0.001, *d* = 0.80]. Concerning reliability, the Cronbach alphas and most of the item-total correlations were very similar in boys and girls, and were lowest for loss of interest in sex and changes in appetite.

**Table 1:** Means, standard deviations (SD) and item-total correlations (r_it_) for the BDI-II items in the total sample (*n* = 835) and for boys (*n* = 345) and girls (*n* = 490).

	**Total sample**		**Boys**		**Girls**	
**BDI-II item**	**Mean (SD)**	**r_****it****_**	**Mean (SD)**	**r_****it****_**	**Mean (SD)**	**r_****it****_**
Sadness (1)	0.95 (0.88)	0.71	0.61 (0.76)	0.63	1.20 (0.87)	0.68
Pessimism (2)	0.88 (0.97)	0.67	0.63 (0.85)	0.61	1.06 (1.00)	0.68
Past failure (3)	1.13 (1.05)	0.70	0.81 (0.97)	0.62	1.35 (1.05)	0.71
Loss of pleasure (4)	1.02 (0.94)	0.70	0.73 (0.84)	0.64	1.22 (0.96)	0.68
Guilty feelings (5)	0.90 (0.93)	0.66	0.62 (0.78)	0.61	1.09 (0.97)	0.64
Punishment feelings (6)	0.83 (1.05)	0.45	0.70 (0.96)	0.44	0.92 (1.10)	0.45
Self-dislike (7)	1.04 (1.12)	0.75	0.57 (0.90)	0.68	1.38 (1.14)	0.72
Self-criticalness (8)	1.13 (1.05)	0.74	0.67 (0.86)	0.66	1.45 (1.05)	0.71
Suicidal thoughts (9)	0.64 (0.80)	0.62	0.39 (0.65)	0.54	0.81 (0.85)	0.60
Crying (10)	0.96 (1.06)	0.66	0.53 (0.93)	0.63	1.27 (1.05)	0.60
Agitation (11)	0.72 (0.84)	0.50	0.55 (0.74)	0.44	0.85 (0.88)	0.49
Loss of interest (12)	0.84 (0.97)	0.68	0.58 (0.83)	0.64	1.02 (1.02)	0.67
Indecisiveness (13)	1.01 (1.02)	0.70	0.68 (0.85)	0.63	1.24 (1.06)	0.69
Worthlessness (14)	0.98 (1.07)	0.76	0.56 (0.85)	0.69	1.28 (1.10)	0.75
Loss of energy (15)	0.95 (0.90)	0.70	0.69 (0.83)	0.63	1.13 (0.91)	0.71
Changes in sleeping (16)	1.25 (0.99)	0.53	1.02 (0.91)	0.50	1.41 (1.01)	0.50
Irritability (17)	1.04 (0.95)	0.54	0.77 (0.89)	0.50	1.22 (0.95)	0.51
Changes in appetite (18)	1.04 (1.00)	0.42	0.82 (0.93)	0.35	1.19 (1.03)	0.41
Concentration difficulties (19)	1.05 (0.96)	0.63	0.84 (0.90)	0.60	1.19 (0.97)	0.64
Tiredness (20)	0.93 (0.90)	0.69	0.67 (0.77)	0.62	1.11 (0.93)	0.69
Loss of interest in sex (21)	0.41 (0.84)	0.33	0.30 (0.72)	0.15	0.48 (0.91)	0.39
Total score/cronbach alpha	19.7 (13.6)	0.94	13.7 (10.9)	0.92	23.9 (13.7)	0.94

For further information, we also provide these descriptive values for the subsample of boys and girls with the diagnosis of depressive disorder (see Supplementary Table 1). Similarly, the girls had consistently higher mean values on each item than did the boys, and this difference was significant with at least *p* < 0.02. The difference in the total score was 8.7 [*t*(469) = 6.89, *p* < 0.001, *d* = 0.70]. Again, the Cronbach alphas and most of the item-total correlations were very similar in boys and girls.

### Goodness of Fit for Proposed Factor Models

Compared to the one-factor model, which already provided acceptable goodness of fit (Table 2), all of the tested simple factor models revealed a better fit. Improvement from the two-factor models to the three-factor models is differential, since the three-factor model of Steer et al. (23) was even lower, whereas the three-factor model of Wu and Huang (28) had the relatively best fit within the simple structure models. Modification indices primarily suggested the inclusion of a residual correlation between guilt and punishment, agitation and irritability, and concentration and agitation, but the expected parameter changes were low (all <0.15); other item pairs, e.g., the three item pairs suggested by Wu and Huang (28), were even less indicated. The factor intercorrelations in all simple factor models were at least > 0.80 and several were > 0.90, providing support for the existence of a general depression factor.

**Table 2:** Confirmatory factor analysis: Fit indices of various factor models suggested in the literature applied to our data.

**Factor models**	**Original sample**	**Chi^**2**^**	**df**	**CFI**	**TLI**	**RMSEA**
**Simple factor models**						
One factor	–	1,093.5	189	0.961	0.957	0.076
Steer et al. (3), 2 factors	Adult outpatients, *n* = 210	790.3	188	0.974	0.971	0.062
Steer et al. (23), 3 factors, 20 items	Adolescent outpatients, *n* = 210	858.1	167	0.970	0.965	0.070
Osman et al. (26), 2 factors	Adolescent inpatients, *n* = 408	836.7	188	0.972	0.969	0.064
Wu and Huang (28), 3 factors	Adolesc., junior high school, Taiwan, *n* = 827	638.3	186	0.981	0.978	0.054
**Second-order model**						
Byrne et al. (14)	Adolescents, non-clinical, Hong Kong, *n* = 487 (twice)	638.3	186	0.981	0.978	0.054
**Bifactor models**						
Ward (7)	Adults, several samples	592.6	174	0.982	0.978	0.054
Osman et al. (16)^a^	Adolescents non-clinical, *n* = 414	531.1	168	0.984	0.981	0.051
Bühler et al. (9)	Adult inpatients, *n* = 569	519.4	165	0.985	0.981	0.051

a *with the MLM estimator [as used in (16)], convergence in model estimation could not be achieved*.

*CFI, comparative fit index; TLI, Tucker–Lewis Index; RMSEA, root-mean-square error of approximation; df, degrees of freedom*.

The application of the second-order model of Byrne (27) to our data did result in a successful solution, but the loading of Performance Difficulties on the second-order factor was very high (0.99) and the loadings of Negative Attitude (0.89) and Somatic Elements (0.92) were also high. Thus, this model was not pursued further, but the results also clearly support the appropriateness of assuming a general factor within a complex factor model.

Concerning the bifactor models, the goodness-of-fit indices favored these models over the two two-factor models with simple structure; however, they did not exceed the model of Wu and Huang (28). Results for the complex factor model of Bühler et al. (9) showed about equal goodness of fit as that for the Ward model and the model of Osman et al. (16).

### Exploratory Factor Analyses

#### Total Sample

The one-dimensional model provided acceptable goodness of fit for the total sample (Table 3), but the fit improved remarkably in the two-factor solution. When allowing for three factors, the increase in goodness of fit compared to the two-factor solution was small. The third factor was dominated by items 11 (agitation) and 17 (irritability), although the loadings were only moderate (0.38 and 0.35, respectively). All other loadings on this third factor were lower than 0.25. Thus, items 11 and 17 shared some further common variance, but did not constitute a factor. Furthermore, factor loadings of factors 1 and 2 of the three-factor solution were very similar to those of the two-factor solution, even for the items 11 and 17.

**Table 3:** Fit indices for exploratory factor analyses with 1–3 factors for the total sample and according to gender.

**Model**	***Chi*^**2**^**	***df***	**CFI**	**TLI**	**RMSEA (90%-CI)**
**Total sample (*****N*** **=** **835)**					
1 factor	1,093.5	189	0.961	0.957	0.076 (0.071–0.080)
2 factors	587.2	169	0.982	0.978	0.054 (0.050–0.059)
3 factors	434.1	150	0.988	0.983	0.048 (0.042–0.053)
**Boys (*****N*** **=** **345)**					
1 factor	501.8	189	0.946	0.940	0.069 (0.062–0.077)
2 factors	303.0	169	0.977	0.971	0.048 (0.039–0.057)
3 factors	243.3	150	0.984	0.978	0.042 (0.032–0.052)
**Girls (*****N*** **=** **490)**					
1 factor	712.7	189	0.959	0.954	0.075 (0.069–0.081)
2 factors	434.4	169	0.979	0.974	0.057 (0.050–0.063)
3 factors	323.2	150	0.986	0.981	0.049 (0.041–0.056)

*CFI, comparative fit index; TLI, Tucker–Lewis Index; RMSEA, root-mean-square error of approximation; df, degrees of freedom*.

The exploratory three factors also lent no support for the three-factor model of Byrne/Wu (20, 27), which provided a good fit in the confirmatory analyses. While the first factor was in good agreement with the Negative Attitude factor of Byrne/Wu (20, 27), our second exploratory factor covered the items of both of Byrne/Wu's factors (20, 27), Performance Difficulty and Somatic Elements, and no item in our third exploratory factor had higher respective loadings than on the second factor. In conclusion, the exploratory factor analyses favored a two-factor solution. The estimated factor loadings are displayed in Table 4.

**Table 4:** Factor loadings for the exploratory two-factor solution for the total sample (*n* = 835) and according to gender (boys *n* = 345; girls *n* = 490).

	**Total sample**		**Boys**		**Girls**	
**BDI-II item**	**F1**	**F2**	**F1**	**F2**	**F1**	**F2**
Sadness (1)	0.59	0.25	0.65		0.46	0.36
Pessimism (2)	0.49	0.31	0.44	0.34	0.50	0.31
Past failure (3)	0.75		0.72		0.76	
Loss of Pleasure (4)	0.32	0.51	0.30	0.50	0.28	0.53
Guilty Feelings (5)	0.78		0.75		0.77	
Punishment feelings (6)	0.64		0.58		0.68	
Self-dislike (7)	0.84		0.75		0.85	
Self-criticalness (8)	0.85		0.74		0.84	
Suicidal thoughts (9)	0.65		0.78		0.51	0.23
Crying (10)	0.53	0.27	0.47	0.36	0.44	0.28
Agitation (11)		0.53		0.57		0.46
Loss of interest (12)	0.16	0.67		0.70	0.19	0.64
Indecisiveness (13)	0.38	0.45	0.23	0.57	0.43	0.38
Worthlessness (14)	0.92		0.94		0.87	
Loss of energy (15)		0.87		0.82		0.88
Changes in sleeping (16)	0.16	0.46		0.60	0.24	0.35
Irritability (17)		0.55		0.55		0.52
Changes in appetite (18)	0.20	0.30		0.39	0.24	0.24
Concentration difficulties (19)		0.73		0.69		0.71
Tiredness (20)		0.90		0.86		0.89
Loss of interest in sex (21)	0.32				0.44	

*only significant values (p < 0.05) are displayed; F1, factor 1; F2, factor 2; Factor intercorrelation: total: r = 0.78; boys: r = 0.72; girls: r = 0.76*.

#### Boys and Girls

A comparison of the one- to three-factor solutions for boys and girls separately revealed very similar results in goodness of fit in both groups (Table 3). The increase from one to two factors was substantial and the addition of further factors did not improve the model fit a great deal. The factor loadings of the two-factor solution according to gender (Table 4) showed the same structure as in the total sample, i.e., the items loaded on the same factor with about the same values, with the exception of the two somatic items 16 (changes in sleeping) and 18 (changes in appetite), as well as 13 (indecisiveness). The two somatic items loaded clearly on factor 2 for the boys, whereas they had similar and lower loadings on both factors for the girls. Indecisiveness loaded on factor 2 for boys and about equally on both factors for girls. Items 1 (sadness) and 9 (suicidal thoughts) loaded on factor 1 in both groups, but seemed to be more pronounced in boys.

In the three-factor solution for the girls, the third factor was determined by items 11 (agitation) and 17 (irritability). Thus, it paralleled the result in the total sample, but the loadings were higher, at 0.55 and 0.60, respectively, and some other items had loadings around 0.30 (concentration, appetite, crying, sadness). On the other hand, several items had loadings of 0.98 and 0.99 on factor one, and one item (worthlessness) had a loading > 1. Thus, this three-factor solution did not seem to be trustworthy. In boys, the third factor was dominated by items 5 (guilt) and 6 (failure), with loadings of 0.47 and 0.37, respectively. All other loadings on this factor were below 0.25 and some were even significantly negative. The loading of item 10 (crying) was not significant. Thus, an interpretation as a guilt/punishment factor (items 5, 6, and 10) as described by Steer et al. (23) was not supported. In line with Steer et al. (23), who judged this factor not to be generalizable, we concluded that items 5 and 6 share some further common variance, but do not constitute a factor. In conclusion, a solution with two factors is the most promising simple structure model in both subgroups, and it is further supported when taking into account the results of Osman et al. (26) and the above-mentioned studies with adults.

### Bifactor Analysis Applied to the Exploratory Two-Factor Model

#### Total Sample

The factor structure derived from the exploratory two-factor model was used to further evaluate the item-factor composition in a confirmatory bifactor model. In this model, all items were required to load on the general factor. The two specific factors (cognitive-affective and somatic) were composed as follows: cognitive-affective with items 1–3, 5–9, and 14; somatic with items 4, 11, 12, 15–17, 19, and 20. Five items were not attributed to a specific factor, i.e., they load only on the general factor: pessimism, crying and indecisiveness due to approximately equal loadings on both factors in the EFA, and changes in appetite and loss of interest in sex due to generally low loadings. The fit estimates for this model were: Chi^2^(173) = 576.03, *p* < 0.001, RMSEA = 0.053, CFI = 0.983, TLI = 0.979; factor loadings are given in Table 5. All items had loadings with *p* < 0.001, with the exception of item 1 (sadness).

**Table 5:** Factor loadings for the confirmatory bifactor solution for the total sample (*n* = 835).

**BDI-II item**	**General factor**	**Cognitive/affective**	**Somatic**
Sadness (1)	0.81	0.07 ^n.s.^	–
Pessimism (2)	0.78	–	–
Past failure (3)	0.75	0.33	–
Loss of pleasure (4)	0.77	–	0.15
Guilty feelings (5)	0.69	0.36	–
Punishment feelings (6)	0.49	0.32	–
Self-dislike (7)	0.80	0.35	–
Self-criticalness (8)	0.78	0.33	–
Suicidal thoughts (9)	0.72	0.15	–
Crying (10)	0.77	–	–
Agitation (11)	0.57	–	0.19
Loss of interest (12)	0.75	–	0.26
Indecisiveness (13)	0.80	–	–
Worthlessness (14)	0.82	0.36	–
Loss of energy (15)	0.75	–	0.42
Changes in sleeping (16)	0.56	–	0.20
Irritability (17)	0.59	–	0.22
Changes in appetite (18)	0.48	–	–
Concentration difficulties (19)	0.68	–	0.33
Tiredness (20)	0.73	–	0.50
Loss of interest in sex (21)	0.45	–	–

*all factor loadings are significant at p < 0.001 except where indicated: n.s., not significant at p = 0.102*.

#### Boys and Girls

The same bifactor model as composed for the total sample was used to test for differences in factor loadings according to gender. Instead of examining these differences in separate factor analyses for boys and girls, the multi-group approach was applied, which also served for testing measurement invariance, described below. The fit estimates of this model were good (cf. M1 in **Table 7**); factor loadings are displayed in Table 6. As in the total sample, all items loaded on the general factor with *p* < 0.001 and with similar values for boys and girls except for item 21 (loss of interest in sex). Factor loadings on the specific factors were also significant at *p* < 0.05 with three exceptions: items 1 (sadness) and 9 (suicidal thoughts) did not load significantly on the cognitive-affective factor for girls while they did so for boys, and the opposite was found for item 4 (loss of pleasure) on the somatic factor.

**Table 6:** Factor loadings for the confirmatory bifactor solution for boys (*n* = 345) and girls (*n* = 490).

	**General factor**		**Cognitive/affective**		**Somatic**	
**BDI-II item**	**Boys**	**Girls**	**Boys**	**Girls**	**Boys**	**Girls**
Sadness (1)	0.73	0.80	0.24	−0.06 ^n.s.^	–	–
Pessimism (2)	0.75	0.78	–	–	–	–
Past failure (3)	0.67	0.76	0.42	0.33	–	–
Loss of pleasure (4)	0.76	0.74	–	–	0.03 ^n.s.^	0.20
Guilty feelings (5)	0.61	0.69	0.48	0.30	–	–
Punishment feelings (6)	0.44	0.51	0.38	0.27	–	–
Self-dislike (7)	0.75	0.77	0.39	0.37	–	–
Self-criticalness (8)	0.71	0.75	0.37	0.33	–	–
Suicidal thoughts (9)	0.62	0.71	0.38	0.02 ^n.s.^	–	–
Crying (10)	0.81	0.69	–	–	–	–
Agitation (11)	0.52	0.56	–	–	0.18^*^	0.15^*^
Loss of interest (12)	0.73	0.74	–	–	0.19	0.29
Indecisiveness (13)	0.77	0.78	–	–	–	–
Worthlessness (14)	0.76	0.81	0.44	0.36	–	–
Loss of energy (15)	0.69	0.74	–	–	0.45	0.44
Changes in sleeping (16)	0.52	0.54	–	–	0.29	0.16
Irritability (17)	0.57	0.54	–	–	0.22	0.20
Changes in appetite (18)	0.42	0.46	–	–	–	–
Concentration difficulties (19)	0.67	0.68	–	–	0.27	0.34
Tiredness (20)	0.67	0.72	–	–	0.53	0.52
Loss of interest in sex (21)	0.24	0.51	–	–	–	–

all factor loadings are significant at p < 0.01 except where indicated:

* *= p < 0.05; n.s., not significant at p < 0.10*.

Taken together, the assumed bifactor structure seems to be superior to a model with two correlated factors, since it is supported not only by the good fit of the model but also by theoretical reasons (assumption of a general depression factor) and the majority of empirical results from other studies. Furthermore, inspection of the modification indices (MI > 30) provided by M*plus* suggest modeling residual correlations in girls for three item pairs (loss of interest and loss of pleasure; agitation and irritability; agitation and concentration) and none for boys, but the modification indices do not suggest relevant changes in factor composition in the two gender groups.

### Tests for Measurement Invariance Across Gender Groups

The bifactor structure derived above (Table 6) served as the baseline model (configural invariance) for testing measurement invariance for boys and girls. The fit of this bifactor composition when modeled as a multi-group CFA was already good (cf. M1 in Table 7), since all three fit criteria (CFI, TLI and RMSEA) indicated good model fit. The results for the constrained model where factor loadings and thresholds are set equal for the two groups are displayed in the second row of Table 7 (M2). The DIFFTEST indicated that adding the loading/threshold invariance did significantly worsen the model fit when compared to the baseline model. Although the three fit indices even became slightly better, at least numerically, and the Chi^2^/df ratio was below the recommended cut-off of 3 (128.5/76 = 1.69), the assumptions of measurement invariance were questioned, and therefore partial measurement invariance was examined. In a first step, the differences in factor loadings of the general factor were evaluated. The only item with a substantial difference was (the already previously identified) item 21 (loss of interest in sex). The constraints for this item were released and the results for this model show (M3 in Table 7) that the DIFFTEST value decreased but was still significant. Next, items on the two specific factors were inspected for differences in factor loadings. As already stated when interpreting Table 6, differences seem to exist in items 1, 4, and 9. In all of these items, the factor loading in one group was significant and substantial, whereas the loading in the other group was not significant. Further releasing the constraints on these three items, the model fit compared to the baseline model was still significantly worse, with *p* < 0.05 (M4 in Table 7). However, it seemed reasonable to refrain from further examinations of item differences at this point. On the one hand, there were no more obvious candidate items with substantial differences in factor loadings between gender groups, and on the other hand, the low Chi^2^/df ratio of 86.0/64 = 1.34 and the good model fit indicated consistently by CFI, TLI, and RMSEA suggested that this model should be accepted as the final model. Nonetheless, the MI indicated further significant improvement when releasing the thresholds 1 and 3 of item 10 (crying). When these two thresholds were additionally set free, the model fit was no longer significantly different compared to the baseline model (*p* = 0.091, M5 in Table 7). The thresholds 1 and 3 were lower for girls in both cases, i.e., the probability of endorsing category 1 instead of category 0 (and 3 instead of 2) was already higher at a lower level of the latent trait. Taken together, it can be concluded that partial measurement invariance holds across the two gender groups.

**Table 7:** Model-fitting results from the measurement invariance tests across boys and girls based on the candidate bifactor model.

**Model**	***Chi*^**2**^**	***df***	**CFI**	**TLI**	**RMSEA**	**DIFFTEST**		
						**value**	***df***	***p***
M1: Configural invariance (no constraints imposed)	713.8	346	0.980	0.976	0.050	–	–	–
M2: Metric invariance (loadings/thresholds equal)	748.8	422	0.982	0.983	0.043	128.5	76	0.0002
M3: Partial metric invariance (item 21 set free)	731.6	419	0.983	0.983	0.042	109.8	73	0.0035
M4: Partial metric invariance (items 1, 4, 9, and 21 set free)	692.6	410	0.985	0.984	0.041	86.0	64	0.0346
M5: Partial metric invariance (as M4, plus threshold 1 and 3 of item 10 set free)	671.6	409	0.986	0.986	0.039	78.5	63	0.0906

*CFI, comparative fit index; TLI, Tucker–Lewis Index; RMSEA, root-mean-square error of approximation; df, degrees of freedom; DIFFTEST against M1*.

A comparison of the latent mean differences between boys and girls revealed a differential result. Since the mean values were set to zero for boys and freely estimated for girls, the latent means of the girls can be tested against zero. The latent mean of the general factor was 0.86 (*p* < 0.001), while the latent means for the specific factors cognitive-affective (0.28, *p* = 0.056) and somatic (−0.12, *p* = 0.362) were not significant. Thus, the difference between boys and girls seems to be almost entirely related to the general factor.

### Tests on Multidimensionality (Total Sample)

The coefficient omega (reflecting all sources of common variance) was 0.96 and omegaH (the reliability of the general factor alone) was 0.88. The omegas for the subscales were also high (0.93 for factor 1 and 0.91 for factor 2) but they shrank considerably to values of 0.12 and 0.14, respectively, when the general factor was partialled out (omegaHS). The relative strength of the general factor as evaluated by the explained common variance (ECV) was calculated as 0.875; the PUC = 0.73. Thus, the combined omegaH, ECV, and PUC indices suggested that the vast majority of variance is accounted for by the general factor and the subscales provide little additional information, although several items have factor loadings > 0.30.

## Discussion

The BDI-II is one of the most commonly used inventories of depression. Given that it is also widely employed in minors, it is important to examine the factor structure in this particular population. Our analyses yielded the following main results: [1] Several factor models proposed in the literature provide a good fit when applied to our data, and differences in goodness of fit are small but in favor of bifactor models; [2] exploratory factor analysis (EFA) revealed that although a one-factor solution already provides an acceptable fit, a two-factor solution with a cognitive-affective and a somatic factor showed a much better fit, both for the total sample and for boys and girls separately; [3] the bifactor model derived from the EFA results improved the goodness of fit further and confirmed the existence of a strong general factor, whereas the loadings of the specific factors cognitive-affective and somatic decreased remarkably but were nevertheless still pronounced; [4] measurement invariance is only partially met: items 21 (loss of interest in sex 21), 1 (sadness), 4 (loss of pleasure) and 9 (suicidal thoughts) show different loadings/thresholds between gender groups, but violations seem negligible.

Before discussing the factor models, the descriptive statistics of our clinical sample of adolescents will be outlined. Taken together, all item-total correlations within the total sample were sufficient to good, with the exception of “loss of interest in sex,” which also showed a low mean value. As our study examined a clinical sample, the mean BDI-II scores were higher compared to those reported in other studies from the general population. Girls had higher (+10.1) mean BDI-II scores compared to boys. For the subsample of adolescents with a diagnosis of depression, the gender difference in mean scores was slightly smaller (+8.7). Moreover, the difference between boys and girls was significant in every item of the BDI-II. Since the mean age was comparable between the two gender groups, it can be assumed that age is not responsible for this difference.

Other studies also found higher mean BDI-II scores in girls than in boys (about five points higher in girls, 23, 26). The effect sizes in our study were moderate to large, and larger than in the samples examined by Osman et al. (26), with *d* = 0.48, and [(23), p. 130], with *d* = 0.38. This finding of gender differences is consistent with the literature on depression, which indicates that girls usually report higher symptoms of depression than do boys [e.g., (30)].

With regard to testing the factor structures of several factor models proposed in the literature, all tested models revealed a good fit. Although the one-factor solution already showed an acceptable fit, the two- or three-factor model revealed a much better fit, although the increase in fit from the two- to the three-factor models was only small. However, the three-factor solution of Wu and Huang (20) revealed the best fit. Additionally, the bifactor models yielded a slightly better fit than the simple factor models, supporting the existence of a general depression factor. Since no model emerged as clearly superior and further explorations seemed necessary, especially for gender differences, EFAs were calculated.

With respect to EFAs, the goodness of fit was again improved by the two-factor solution compared to the already acceptable fit of the one-factor model. Our study indicated a similar factor structure to the two-factor solution (“cognitive-affective”; “somatic-affective”) of Osman et al. (26) and Uslu et al. (21), who also examined adolescent psychiatric inpatients, but the item loadings in our study were somewhat different. Exploratory solutions with three and with four factors revealed better goodness-of-fit indices, but the item attribution was not satisfactory: The third factor in the three-factor EFA had no loadings that were higher than the respective loadings on factor 2 (see results section for details) and the three-factor model of Wu and Huang (28), which provided good fit in the confirmatory analyses, was not supported. Concerning the four-factor solution, the fourth factor – labeled “guilt/punishment” (items 5, 6, and 10) by Steer et al. (23) – could not be supported, as the item “crying” did not load significantly. Furthermore, fewer than three items do not really constitute a factor, which is why the four-factor solution seems to be the least acceptable one despite showing the highest goodness-of-fit indices. Taken together, our exploratory factor analyses favor the two-factor solution: Factor 1 can be titled “cognitive-affective” and factor 2 “somatic.” This is in line with the US manual for the BDI-II and with several international studies (1, 4, 26). Furthermore, the two-factor solution of Osman et al. (26) showed measurement invariance across different ethnic groups (African American, Hispanic, and Caucasian) of adolescent inpatients (41).

Concerning the factor loadings, most items loaded with respect to content on either the factor “cognitive/affective” (e.g., pessimism, guilty feelings, self-dislike) or the factor “somatic” (e.g., agitation, loss of energy, tiredness). However, some items loaded non-intuitively: For example, item 10 (crying) loaded more strongly on the factor “cognitive/affective” in our sample, despite loading on the “somatic factor” in most other studies (17, 21, 23, 25). Nevertheless, this item also loaded on the “cognitive factor” in the study by Wu and Chang (20) and on both the “cognitive” and the “somatic factor” in the study by Asal and Abdel-Fattah (19). Moreover, item 4 (loss of pleasure) and item 13 (indecisiveness) loaded on different factors across different studies. These differing classifications may be attributable to differences in the study populations and age groups assessed in the various studies. With regard to the present data, item 21 (loss of interest in sex) showed a small mean value and low reliability. Furthermore, it had a low factor loading in the one-factor solution, varying from 0.31 to 0.34. This resembles the results of Huang and Cheng (4) with respect to the one-factor solution (0.28) and two- and three-factor models (0.29–0.30) as well as the study by Wu and Huang (28), who found a low factor loading (0.36) on the factor “Somatic Elements.” The loading of 0.76 on the general factor in the study by Osman et al. (16) appears to be an outlier and is rather dubious given the low inter-item correlations between item 21 and all other items.

Factor loadings split by gender in our sample were comparable to those of the whole sample. However, item 21 (loss of interest in sex) loaded on the somatic factor in the total sample and on the cognitive factor when split by gender. Item 21 primarily loaded on the “cognitive factor” in other studies (19, 20, 25) - also when split by gender (19). Interestingly, in girls, item 16 (changes in sleeping) and item 18 (changes in appetite) loaded on both factors rather than only on “somatic affective,” while both of these items loaded on the “somatic factor” in the majority of previous studies (15, 17, 18, 21, 23), mainly also when split by gender (15, 18, 26).

It has been argued that a refinement of some items should be considered, especially regarding item 10 (crying) and item 21 (loss of interest in sex), as it can be questioned whether these items are good markers of depression in minors or whether they should be revised or even deleted in future versions of the BDI-II. Item 10 (crying) has been found to be problematic in many studies and often shifts across the factors. In our study, however, the factor loadings were strong on the general factor and essentially comparable between the two gender groups, but there were hints that the thresholds may be different, i.e., girls endorse the respective category earlier than do boys. Concerning item 21 (loss of interest in sex), which had low to insufficient factor loadings in almost all studies cited above, the evaluations by expert raters in the study by Osman et al. (26) also revealed a low relevance and specificity of this item. The adolescents themselves also found that the item was not very useful, but evaluated the formulation and clarity of the item to be as high as for other items. Thus, revising the text may be neither necessary nor sufficient to improve the measurement ability of this item. It seems that item 21 is not relevant for adolescents, but as it has little negative effect on reliability, it should be kept for reasons of comparison with other samples.

The bifactor model derived from the exploratory factor analyses showed good fit and confirmed the proposed factor composition, since the modification indices suggested no further substantially different attribution of items. All items loaded strongly on the general factor, and the items attributed to the two specific factors also had significant loadings, with the exception of item 1 (sadness). Sadness belongs clearly to the general depression factor, and disappears on the specific factor cognitive/affective, although it remains significant (but low) in girls. Other items with ambiguous loadings on the two EFA factors were also captured by the general factor, e.g., 4 (loss of pleasure), 10 (crying), or 11 (indecisiveness). The specific factor cognitive/affective is mainly defined by items 3, 5–8, 14, and thus merely cognitive. The specific factor somatic is dominated by items 15 (loss of energy), 20 (tiredness), and (to a lesser degree) 19 (concentration difficulties). Item 12 (loss of interest) also loads on the specific factor somatic, although it is often related to the cognitive/affective factor [e.g., (16)], and thus seems to have a predominantly somatic component in adolescents.

Concerning gender differences in factor loadings, a difference emerged for item 21 (loss of interest in sex) on the general factor. All other differences on the general factor were small (<0.12). With regard to differences on the specific factors, differences were also minimal, with the exception of items 1 (sadness), 4 (loss of pleasure) and 9 (suicidal thoughts). The tests on measurement invariance recommended freeing the factor loadings of these four items; hence, partial measurement invariance could be achieved. However, the already good fit of the unrestricted model and the minor differences in factor loadings suggested that violations in measurement invariance are negligible. Osman et al. (26) also noted that only minor differences in factor loadings were observed (but without performing a formal test). Within an item response theory (IRT) context, de Sá et al. (42) analyzed differential item functioning (DIF) in a large Brazilian college student sample, and found DIF in gender for item 10 (crying) and DIF in age for item 21 (loss of interest in sex). However, the age range of the students was somewhat higher (16–30 years) for their young subgroup (the subgroup of older participants was > 30 years of age) than in the sample analyzed in the present study.

Given the good fit of the bifactor model and only minor violations in measurement invariance, the bifactor statistical indices were calculated for the bifactor solution of the total sample. These indices were clearly in favor of considering the BDI-II as a unidimensional scale, and these findings are in line with the results of studies in adult samples (the respective information for adolescent samples is not yet available). Brouwer et al. (8) found that the general factor in the Ward model accounted for 77% of the common variance, while the cognitive and the somatic factor explained 8 and 15%, respectively. Similar results were reported by McElroy et al. (12), with ECV = 0.69, and Lim et al. (2019), with ECV = 0.81. Our own ECV = 0.875 (for adolescents) even exceeds these values. Thus, these values indicate that there is no need to model the BDI-II items in a full bifactor measurement model within a structural equation modeling approach (c.f. 6). In terms of coefficient omega hierarchical (ω_H_), we found 0.88 for the general factor alone and 0.12 and 0.14 for the two specific factors alone (ω_HS_). The corresponding values in the adult samples were ω_H_ = 0.84 (12) and ω_H_ = 0.89 (13) for the general factor alone, ω_HS_ = 0.06; 0.01; 0.01 (12) for the three specific factors alone, and ω_HS_ = 0.18; 0.30 for the two specific factors alone (13). All of these indices strongly support the assumption that the BDI-II reflects a unidimensional scale. Thus, one should be cautious when interpreting subscale scores, because these scores provide little additional information. Furthermore, they are highly related to the general construct and should only be used in conjunction with the general score (8, 12).

Huang and Cheng (4) concluded that the finding of a general depression factor does not invalidate the differentiation of specific depression sub-factors, which in turn might be related to different etiologies and external criteria. However, the authors did not analyze bifactor models (the correlation between the general factor and one of the depression subfactors was not identified in some models; nevertheless, it remains unclear why they did not fix it to zero, as should be the case in bifactor models).

Beck and colleagues themselves developed a reduced BDI-II version [BDI Fast Screen - BDI-FS, (43)] in which the somatic items were dropped. This could be used, for instance, to measure and filter depressive symptoms in patients with mainly somatic symptoms, whose BDI-II scores might be automatically high due to high values on the somatic subscale, e.g., in cardiology.

From a clinical perspective, we would like to add that even single items can be useful in diagnostics, e.g., the items 9 (suicidal thoughts) or 16 (changes in sleeping). Responses to item 9 (suicidal thoughts) have been shown to be in good agreement with a clinician rating of suicidal behavior (44), and this information suggests that it may be valid to explore suicidal tendencies via self-report.

## Limitations

The item-factor composition in our confirmatory bifactor model was deduced from exploratory factor analysis and should therefore be replicated in an independent sample, especially to confirm the four items found to be responsible for gender differences in factor loadings. Furthermore, bifactor models were criticized for their tendency to show superior goodness of fit in model comparison studies (45). However, bifactor models are not necessarily favored in factor analyses of presumably multidimensional instruments (46, 47), and our preference for a bifactor model was not only based on the results of previous studies with model comparisons based on fit indices, but also on the high factor correlations > .80 that were found in almost all simple factor models. In addition, and following a distinction made by Bonifay et al. (45), our bifactor model is not intended to represent a structure of depression psychopathology, but solely for reflecting psychometric properties, i.e., to inform about the degree to which the BDI-II yields an univocal total score and the extent to which the subscales yield reliable scores after accounting for the general factor. Another way to address unidimensionality, and in order to avoid this type of methodological problems along with the difficult interpretability of the specific factors in bifactor models (6), may be in the application of models from item response theory (IRT) (48, 49). Most of these studies, however, usually drop less-fitting items and result in shorter versions of the instrument. Interestingly, a study (50) combining 12 data sets from clinical and non-clinical adolescent samples (yielding a total of 3,403 participants) that applied IRT modeling to ten core depression symptoms obtained in a clinical interview found that they were all highly discriminating indicators of depression and they were “remarkably unidimensional” [(50), p. 827].

The strengths of the current study lie in the large sample of adolescent patients, a substantial proportion of whom had an F3 diagnosis. A limitation is the heterogeneity of the sample, which was a mixed sample of inpatients and outpatients with different comorbidities. Furthermore, the diagnosis was not validated with a clinical/structured interview. Thus, further studies should use clinical interviews to increase the reliability of the diagnosis.

## Conclusion

Taken together, the results indicated that both the bifactor and the two-factor model yielded satisfactory solutions for adolescents with various psychiatric disorders and for adolescents suffering from depressive symptoms. A bifactor model, however, seems preferable, and the bifactor statistical indices suggested that the BDI-II primarily reflects a unidimensional scale. It can be concluded that for practical reasons, the total score of the BDI-II can be used for measuring depression severity in clinical settings. If more detailed analyses are necessary, it might be reasonable to additionally analyze subscales.

## Data Availability Statement

The datasets generated for this study are available on request to the corresponding author.

## Ethics Statement

Ethical review and approval was not required for the study on human participants in accordance with the local legislation and institutional requirements. Written informed consent to participate in this study was provided by the participants' legal guardian/next of kin.

## Author Contributions

JS and FK designed the study and wrote the first draft of the manuscript. FK conducted the statistical analyses. All authors read, approved the final version of the manuscript, managed the literature searches, analyses, and carried out the data preparation.

## Conflict of Interest

FK was a recipient of royalties (as one of three co-authors) from the German version of the BDI-II. The remaining authors declare that the research was conducted in the absence of any commercial or financial relationships that could be construed as a potential conflict of interest.
