# Supplementary material for: Factor Structure and Measurement Invariance Across Gender of the Beck Depression Inventory-II in Adolescent Psychiatric Patients
Source: Front Psychiatry. 2020 Dec 23;11:527559. doi: 10.3389/fpsyt.2020.527559 (PMC7785793; doi:10.3389/fpsyt.2020.527559)
Supplement: Supplementary file 1 [file Table_1.DOC]

Supplementary Table 1: Means, standard deviations (SD) and item-total correlations (r_it_) for the BDI-II items in the sample with a diagnosis of depression: total sample (*n* = 471), boys (*n* = 142), and girls (*n* = 329).

|  | Total sample | |  | Boys | |  | Girls | |
| --- | --- | --- | --- | --- | --- | --- | --- | --- |
| BDI-II item | mean (SD) | r_it_ |  | mean (SD) | r_it_ |  | mean (SD) | r_it_ |
| Sadness (1) | 1.28 (0.88) | .66 |  | 0.95 (0.87) | .63 |  | 1.42 (0.84) | .63 |
| Pessimism (2) | 1.22 (0.99) | .63 |  | 1.01 (0.95) | .60 |  | 1.30 (0.99) | .63 |
| Past Failure (3) | 1.47 (1.00) | .65 |  | 1.20 (0.96) | .60 |  | 1.58 (1.00) | .65 |
| Loss of Pleasure (4) | 1.28 (0.96) | .65 |  | 0.98 (0.94) | .64 |  | 1.42 (0.94) | .63 |
| Guilty Feelings (5) | 1.13 (0.98) | .62 |  | 0.87 (0.87) | .59 |  | 1.24 (1.00) | .61 |
| Punishment Feelings (6) | 0.93 (1.10) | .44 |  | 0.75 (1.05) | .48 |  | 1.01 (1.11) | .41 |
| Self-Dislike (7) | 1.40 (1.13) | .69 |  | 0.88 (1.03) | .68 |  | 1.63 (1.09) | .65 |
| Self-Criticalness (8) | 1.46 (1.03) | .71 |  | 0.98 (0.96) | .70 |  | 1.67 (1.00) | .67 |
| Suicidal Thoughts (9) | 0.87 (0.83) | .55 |  | 0.63 (0.75) | .52 |  | 0.97 (0.84) | .53 |
| Crying (10) | 1.23 (1.06) | .61 |  | 0.77 (1.02) | .64 |  | 1.43 (1.01) | .55 |
| Agitation (11) | 0.85 (0.88) | .47 |  | 0.66 (0.78) | .43 |  | 0.94 (0.91) | .46 |
| Loss of Interest (12) | 1.12 (1.01) | .65 |  | 0.85 (0.92) | .66 |  | 1.24 (1.02) | .63 |
| Indecisiveness (13) | 1.31 (1.04) | .67 |  | 0.99 (0.99) | .61 |  | 1.44 (1.04) | .66 |
| Worthlessness (14) | 1.36 (1.07) | .72 |  | 0.94 (0.97) | .70 |  | 1.54 (1.06) | .69 |
| Loss of Energy (15) | 1.25 (0.92) | .67 |  | 0.99 (0.94) | .57 |  | 1.36 (0.89) | .68 |
| Changes in Sleeping (16) | 1.49 (0.99) | .47 |  | 1.32 (0.97) | .43 |  | 1.56 (1.00) | .47 |
| Irritability (17) | 1.21 (0.96) | .51 |  | 0.87 (0.93) | .53 |  | 1.36 (0.93) | .45 |
| Changes in Appetite (18) | 1.19 (1.03) | .36 |  | 1.00 (0.94) | .30 |  | 1.27 (1.05) | .35 |
| Concentration Difficulties (19) | 1.25 (0.95) | .63 |  | 1.01 (0.93) | .64 |  | 1.35 (0.94) | .60 |
| Tiredness (20) | 1.20 (0.94) | .66 |  | 0.93 (0.84) | .61 |  | 1.32 (0.95) | .65 |
| Loss of Interest in Sex (21) | 0.44 (0.87) | .39 |  | 0.29 (0.67) | .41 |  | 0.50 (0.94) | .37 |
| Total score / Cronbach alpha | 24.9 (13.2) | .93 |  | 18.9 (12.0) | .92 |  | 27.6 (12.8) | .92 |
